# Emoji-Driven Sentiment Analysis for Social Bot Detection with Relational Graph Convolutional Networks

**DOI:** 10.3390/s25134179

**Published:** 2025-07-04

**Authors:** Kaqian Zeng, Zhao Li, Xiujuan Wang

**Affiliations:** College of Computer Science, Beijing University of Technology, Beijing 100124, China; 22013220@emails.bjut.edu.cn (K.Z.); lizhao@emails.bjut.edu.cn (Z.L.)

**Keywords:** social bot detection, emoji, sentiment analysis, deep learning, RGCN

## Abstract

The proliferation of malicious social bots poses severe threats to cybersecurity and social media information ecosystems. Existing detection methods often overlook the semantic value and emotional cues conveyed by emojis in user-generated tweets. To address this gap, we propose ESA-BotRGCN, an emoji-driven multi-modal detection framework that integrates semantic enhancement, sentiment analysis, and multi-dimensional feature modeling. Specifically, we first establish emoji–text mapping relationships using the Emoji Library, leverage GPT-4 to improve textual coherence, and generate tweet embeddings via RoBERTa. Subsequently, seven sentiment-based features are extracted to quantify statistical disparities in emotional expression patterns between bot and human accounts. An attention gating mechanism is further designed to dynamically fuse these sentiment features with user description, tweet content, numerical attributes, and categorical features. Finally, a Relational Graph Convolutional Network (RGCN) is employed to model heterogeneous social topology for robust bot detection. Experimental results on the TwiBot-20 benchmark dataset demonstrate that our method achieves a superior accuracy of 87.46%, significantly outperforming baseline models and validating the effectiveness of emoji-driven semantic and sentiment enhancement strategies.

## 1. Introduction

Social media platforms such as Twitter (now renamed X) and Facebook have emerged as pivotal channels for global users to access news, engage in public discourse, and foster emotional interactions, owing to their real-time dissemination capabilities and low-barrier communication mechanisms [1]. According to Meta Group’s financial reports [2], its monthly active users surged exponentially from 1.5 billion in 2015 to 3 billion in 2023. However, this thriving social ecosystem coexists with the rampant proliferation of malicious social bot accounts. A study by the University of Southern California revealed that 15% of Twitter accounts are bots [3], which are automated programs designed to perform social media activities [4], with 78.6% implicated in disseminating disinformation [5]. These bots mimic genuine user behaviors to establish a systematic attack chain: “fabricating pseudo-consensus → inducing group polarization → manipulating decision-making processes” [6]. A notable example occurred during the 2016 U.S. presidential election, where the Botometer system [7] identified over 280,000 bot accounts persistently spreading political misinformation, accounting for one-third of candidate-endorsing tweets [8]. Such activities distorted voter perceptions and severely undermined electoral integrity. Therefore, the detection of social bots has attracted the attention of relevant researchers.

Current mainstream detection methodologies face multifaceted technical challenges. First, their capability to identify highly anthropomorphic behaviors remains limited; social bots now emulate human-like information propagation patterns, significantly degrading the performance of detection models reliant on static shallow statistical features [9]. Second, bots increasingly exploit social network analysis techniques to construct and optimize their relational networks, rendering their interactions indistinguishable from authentic users [10]. These technological advancements have made existing detection methods ineffective in identifying malicious bot accounts, thus creating an urgent demand for a new detection framework to tackle this challenge.

**Figure 1:**
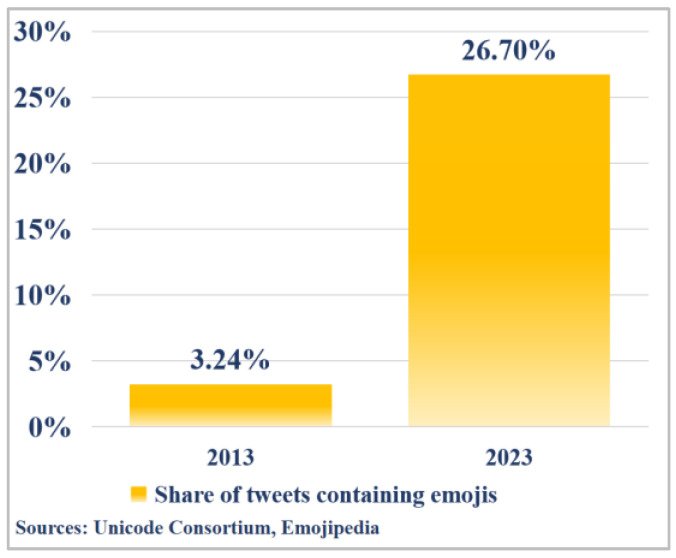
Emoji use in tweets (2013 vs. 2023).

In social bot detection, tweets represent one of the most critical feature sources. However, most existing detection methods dismiss emojis as noise and routinely eliminate them during preprocessing [11]. However, in reality, emojis not only significantly enhance the semantic interpretability of tweets but also provide emotional cues absent in plain text [12,13,14,15,16]. According to a report from Emojipedia, the proportion of tweets containing emojis on Twitter has surged from 3.24% in 2013 to 26.7% in 2023 [17]. As illustrated in Figure 1, emoji usage frequency increased by 724% over this decade, underscoring their growing role as core carriers of nuanced semantics and affective signals. Taking the tweet “Let’s enjoy some 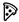 and watch 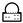 at the 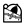 later!” as an example, when analyzed solely through its textual content, the meaning becomes ambiguous. However, integrating emojis with the text resolves semantic uncertainties, revealing a clear contextual narrative. This contrast demonstrates that emojis encode rich semantic information, thereby proving their irreplaceable value in tweet interpretation.

**Figure 2:**
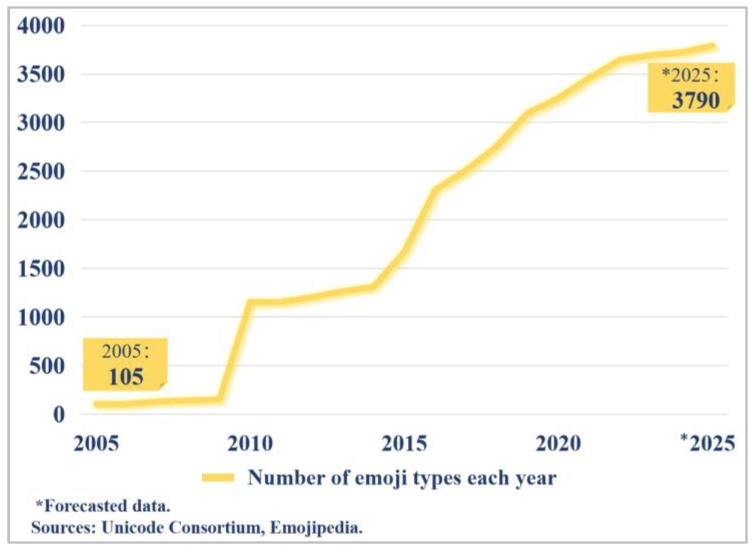
Growth of emoji types (2005–2025).

Beyond semantic enrichment, emojis excel at conveying emotional information that text alone often fails to capture. Particularly in informal short texts such as comments, they serve as critical indicators of user sentiment. Taking the tweet “It’s raining on my picnic day. 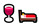
” as an example, if the emojis are ignored, it expresses the negative emotions of disappointment and chagrin after the plan is ruined. However, after adding the “
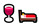
” emojis, the sentiment of the sentence undergoes a reversal, conveying an easygoing and adaptable attitude of “Since it’s raining and the picnic can’t happen, might as well have a drink and then go to sleep”, thus transforming into a positive sentiment. To meet escalating demands for emotional articulation, the Unicode Consortium has expanded emoji categories through iterative updates. As illustrated in Figure 2, official data reveal a 35-fold increase in emoji diversity from 2005 to 2025 [18], with an annual growth rate of 42.7%. This expansion substantially elevates emojis’ research value in sentiment analysis.

To address the aforementioned challenges, we propose ESA-BotRGCN, an emoji-driven multi-modal detection framework, with its core innovations spanning five key dimensions:

1. We utilize the Emoji Library to convert emojis into textual descriptions, refine the coherence of transformed tweets via GPT-4, and generate semantically and sentiment-enriched tweet embeddings using RoBERTa.
2. We propose a seven-dimensional sentiment feature quantification framework, leveraging foundational sentiment metrics, including positive, negative, and neutral sentiment values and complexity measures, to further capture statistical discrepancies between bot accounts and genuine users in terms of sentiment polarity span, dynamic volatility, and expression consistency.
3. An attention gating mechanism is developed to adaptively integrate sentiment features, user descriptions, tweet content, numerical attributes, and categorical features into a globally context-aware unified feature representation.
4. To capture bot-specific behavioral patterns, a Relational Graph Convolutional Network (RGCN) is constructed to model user-following relationships based on graph topological structures.
5. We conduct extensive experiments on the TwiBot-20 and Cresci-15 datasets, demonstrating our model’s superiority through significant improvements in accuracy, F1-score, and MCC over mainstream baselines.

## 2. Related Work

Research in social bot detection has evolved over decades, primarily categorized into four methodological paradigms: Content-based methods analyze lexical distributions, metadata (e.g., URL ratios), and basic statistical patterns in user-generated text to build detection models. Behavior-based methods focus on identifying anomalies in temporal user activity patterns (e.g., posting frequency, active hours) or interaction behaviors (e.g., retweet/reply chains). Deep learning approaches leverage neural architectures like Convolutional Neural Networks (CNNs) and Recurrent Neural Networks (RNNs) to automatically extract deep features from user content, significantly enhancing detection performance in complex scenarios. Graph neural network (GNN)-based methods model user social relationship networks (e.g., follow/retweet graphs), employing Graph Convolutional Networks or Graph Attention Networks to capture latent structural correlations. The following sections systematically review the core advancements and technical characteristics of these approaches.

### 2.1. Content-Based Approaches

Content-based social bot detection methods primarily rely on analyzing statistical patterns, metadata, and linguistic styles of user-generated text to construct classification models. Early studies extracted shallow features such as keyword frequency, URL ratio, and special character usage and combined these with machine learning algorithms for preliminary detection.

Sivanesh et al. [19] proposed a multidimensional feature-based approach for identifying social bots. This method integrates both account attributes and behavioral features to distinguish bots from real users. Specifically, they treat user ID and account creation time as core features while also incorporating auxiliary features such as the posting time and source of the user’s most recent 50 tweets. A probabilistic mathematical model is then used to calculate the likelihood that a user is a bot, enabling precise discrimination between bots and genuine users. Kantepe et al. [20] utilized accounts suspended by Twitter (now renamed X), extracting 62 different features by analyzing tweets, profile information, and temporal behaviors. These features were then used for classification with logistic regression (LR), multinomial Naive Bayes, support vector machines (SVM), and gradient boosting (GB). ESC [21] extracted over 100 feature indicators by combining three groups of features: user attributes, Twitter behavioral data, and textual features. Dedicated classifiers were trained for each type of bot, and their predictions were aggregated using a max-rule strategy.

While content-based approaches have achieved initial success in bot detection, their core limitation lies in the over-reliance on static and shallow features, coupled with a systematic neglect of the semantic and sentiment values embedded in emojis. Methods such as those proposed by Sivanesh [19] and Kantepe [20], despite extracting extensive metadata and text statistical features, commonly discard emojis as noise during preprocessing. This practice results in the loss of critical semantic cues and sentiment expression patterns, significantly undermining their effectiveness against modern, highly anthropomorphic bots capable of dynamic evasion. Moreover, their reliance on manually engineered features restricts generalization capabilities when faced with rapidly evolving bot strategies, struggling to capture deeper statistical disparities such as sentiment complexity and polarity span. Research by Grimme et al. [22] indicates that in several mature detection tools, features like keyword frequency and timing contribute minimally to detection performance. Due to their simplicity and regularity, these features can be easily learned and mimicked by bot developers, allowing bots to evade detection by adjusting such attributes [23]. In contrast, ESA-BotRGCN fundamentally addresses these issues through innovative emoji–text mapping and GPT-4 coherence optimization. By generating tweet embeddings rich in semantic and sentiment information using RoBERTa and designing seven-dimensional sentiment features, our method not only preserves emoji information but also captures more nuanced and discriminative user behavioral patterns.

### 2.2. Behavior-Based Approaches

Although content-based methods demonstrated promising results in early studies, their reliance on manually engineered features limits their generalization capability. To address this limitation, researchers have shifted toward in-depth exploration of user behavioral patterns, thereby establishing a behavior-based detection paradigm. These approaches aim to distinguish social bots from real users by analyzing user activity patterns and interaction behaviors on social platforms to identify anomalies. The central assumption is that bot accounts exhibit statistically measurable deviations in their temporal behaviors (e.g., posting intervals, activity cycles) and interactive behaviors (e.g., retweeting, commenting chains).

In the work by Ruan et al. [24], user behaviors were categorized into outward and inward actions based on their influence on others. By computing the variance of these behaviors, the authors estimated the likelihood of an account being compromised, enabling effective detection of hijacked accounts. Amato et al. [25] modeled behavioral sequences—such as logins, message posts, and photo sharing—using Markov chains to capture typical behavioral patterns of real users. Deviations from these patterns were then used to identify anomalies, effectively detecting social bots. Cresci et al. [26,27] proposed a novel unsupervised detection method that encodes user behavior sequences along a timeline into DNA-like strings, where each type of tweet is mapped to a nucleotide: original tweet → A, retweet → C, and reply → T. The similarity between these behavioral “DNA sequences” is then measured using the Longest Common Subsequence (LCS) algorithm, and similarity scores are used to differentiate between real users and bots. Peng et al. [28] introduced UnDBot, an unsupervised detection framework grounded in the principles of structural information theory. This method leverages the structural properties of social networks and performs community detection by minimizing structural entropy. It is particularly effective at identifying bot clusters engaged in coordinated attacks. This study further explores the applicability and robustness of the method across social networks of varying sizes and types.

Behavior-based methods identify anomalies through temporal sequences and interaction patterns, circumventing some limitations of content analysis. However, their primary weakness lies in the complete stripping away or superficial utilization of the semantic content of tweets. For instance, Cresci’s pioneering “DNA sequence” method [26,27], while effective in capturing anomalies in behavioral sequences, entirely neglects the semantic information and sentiment tendencies embedded in tweet texts (including emojis). Peng’s UnDBot [28], focused on structural community detection, underutilizes individual-level content features such as sentiment expression. Such methods may fail against bots that closely mimic human behavior patterns but exhibit statistical deviations in sentiment expression (e.g., low sentiment volatility and excessively high consistency). In contrast, ESA-BotRGCN overcomes this limitation by deeply fusing content with behavioral/topological information: it not only models social relationship topologies using RGCN but, crucially, takes semantically and sentimentally enhanced tweet content and specially designed seven-dimensional sentiment features as core inputs. Through an attention mechanism, it dynamically fuses these with behavioral/structural features, enabling joint modeling of users’ multi-dimensional abnormal patterns and achieving stronger discriminative capabilities.

### 2.3. Deep Learning-Based Approaches

To overcome the limitations of traditional methods that heavily rely on feature engineering, deep learning techniques have enabled a qualitative leap in detection capabilities through end-to-end learning. Deep learning-based social bot detection methods automatically extract high-level semantic and behavioral features via neural networks, significantly improving performance in complex scenarios. The core strength of such methods lies in their ability to bypass the limitations of traditional manual feature engineering by directly learning discriminative patterns from raw data.

Ilias et al. [29] proposed a deep learning model that integrates an embedding layer, bidirectional LSTM, and dense layers, and was among the first to introduce an attention mechanism into the context of social bot detection. In 2020, Wu et al. [30] introduced a novel social spammer detection method based on xDeepFM, which extracted 30 features from multiple dimensions, including user behavior, content, and social relationships. The xDeepFM model was then employed to model these features for identifying malicious users on the Sina Weibo platform. In 2021, Wu et al. [31] further developed an innovative detection framework named DABot. This framework utilizes a residual network to mitigate the vanishing gradient problem in deep neural network training, thereby enhancing training efficiency and model stability. Additionally, a bidirectional gated recurrent unit (BiGRU) was introduced to capture bidirectional dependencies in user behavior sequences, enabling more accurate modeling of user activity patterns. The framework also incorporates an attention mechanism to emphasize critical features and enhance the model’s sensitivity to important information. Arin et al. [32] proposed a deep learning architecture consisting of three Long Short-Term Memory (LSTM) models and a fully connected layer to capture the complex activity patterns of humans and bots on social media. Due to the architecture involving the integration of multiple components across different hierarchical levels, the authors explored three distinct training strategies to effectively optimize each component. Fazil et al. [33] presented a deep neural network model, DeepSBD, which performs comprehensive multimodal modeling—including user profiles, temporal sequences, activity patterns, and content information—for efficient detection of bot accounts. This model employs a two-layer stacked BiLSTM architecture to jointly model user timelines and behavioral patterns, while a Deep Convolutional Neural Network (CNN) is used to extract semantic features from content. Furthermore, an attention mechanism is integrated to dynamically optimize the feature fusion process, significantly enhancing model discrimination in complex scenarios. Najari et al. [34] proposed a GAN-based detection framework, GANBOT, which introduces LSTM layers as a shared module between the generator and discriminator. This design not only addresses the convergence issues commonly associated with traditional SeqGAN in text generation tasks but also enables the classifier to directly access enriched information regarding bot behavior patterns from the generator, thereby providing a novel technical paradigm for social bot detection. Feng et al. [35] used three state-of-the-art large language models (LLMs), including Mistral-7B, LLaMA2-70B, and ChatGPT, for direct social bot detection. On the TwiBot-20 dataset, the accuracies were 60.9%, 66.1%, and 63.2%, respectively. LLMs bring new opportunities for social bot detection, yet their performance still needs to be improved.

Deep learning methods have achieved remarkable progress in automatic feature learning, surpassing traditional manual feature engineering. However, they still have significant deficiencies in handling the semantic and sentiment values of emojis and in efficiently fusing multimodal heterogeneous features. Most existing models usually directly remove emojis or only perform simple replacements during the text preprocessing stage, failing to fully tap their potential as crucial sentiment carriers. Although LLM-based methods [35] have shown promise, their accuracy in social bot detection is still far lower than that of specialized models, and they incur high computational costs. In contrast, the innovations of ESA-BotRGCN lie in the following: first, specially designing a process for semantic enhancement of emojis and quantification of sentiment features, ensuring that deep models can make full use of emoji information; and second, introducing an attention-gating mechanism to dynamically learn and fuse sentiment features, text content, user attributes, numerical/categorical features, and graph-structure information, significantly improving the effectiveness of multimodal information fusion and the discriminative ability of the model.

### 2.4. Graph Neural Network-Based Approaches

Unlike deep learning methods that focus primarily on textual analysis, graph neural networks (GNNs) are designed to capture collective behavioral patterns from social relational networks, making the two approaches complementary in modality. These methods model the topological structure of users’ social networks to detect anomalous interaction patterns associated with bot accounts. A key advantage of GNNs lies in their ability to directly process non-Euclidean data (e.g., follower or retweet graphs) and to reveal hidden structural features through neighborhood information aggregation via message-passing mechanisms.

In 2021, Feng et al. [36] introduced BotRGCN, marking the first application of RGCN to the modeling of relational heterogeneous graphs. By capturing multi-type interactions among users—such as follows and retweets—they constructed a heterogeneous information network in which users serve as nodes and various interaction types as edges, thereby enhancing account detection performance. In 2022, Feng et al. [37] further enhanced the model architecture by introducing a relational graph Transformer module, which leverages self-attention mechanisms to dynamically model heterogeneous influences between users, thereby addressing the traditional RGCN’s limitation in capturing complex relational dependencies. Li et al. [38] proposed SybilFlyover, a detection model based on heterogeneous graphs for identifying fake accounts. This approach represents the complex interactions among multi-type entities using a directed heterogeneous social network graph. A prompt learning strategy is employed to inject content-based social information into the model for enhanced state modeling. Finally, transformer-based mechanisms are used to process the social graph and identify nodes indicative of Sybil accounts. Peng et al. [39] proposed a Domain-Aware Multi-Relational Graph Neural Network (DA-MRG), which constructs a multi-relational graph based on user features and interaction types to generate graph embeddings and employs a domain-aware classifier to distinguish bot accounts. To address the behavioral similarities of bots across different social networks, they designed a joint learning framework that enables privacy-preserving cross-platform data sharing, thereby enhancing detection performance. Wang et al. [40] proposed an unsupervised social bot detection method, BotDCGC, based on deep contrastive graph clustering. The method employs a graph attention encoder to extract node embeddings by integrating account features with topological structure information and utilizes an inner product decoder to reconstruct the network. Structural contrastive learning is used to enhance the discriminability of node embeddings, while similarity-based calculations generate high-confidence pseudo-labels that dynamically guide the joint optimization of embedding learning and clustering, thereby improving detection performance.

Graph neural network (GNN) methods have demonstrated significant advantages in capturing group behavioral patterns and detecting coordinated bots by explicitly modeling social relationship topologies. However, existing GNN approaches generally suffer from two limitations: First, they underutilize or coarsely fuse the rich content/attribute features of nodes. Their node representations are relatively simplistic and fail to fully integrate semantically and sentimentally enhanced tweet content, user descriptions, and specially quantified high-order sentiment features [41]. Second, most methods construct homogeneous graphs, overlooking the extraction of heterogeneous topological graphs containing diverse social relationships. In contrast, ESA-BotRGCN achieves core breakthroughs by the following: first, using highly informative node features as the input foundation for RGCN; and second, constructing a heterogeneous social topology graph, enabling ESA-BotRGCN to simultaneously leverage both the multimodal intrinsic attributes of nodes and their social topological context information—particularly effective in scenarios with diverse social relationships—thereby enhancing detection accuracy.

## 3. ESA-BotRGCN Model Architecture

**Figure 3:**
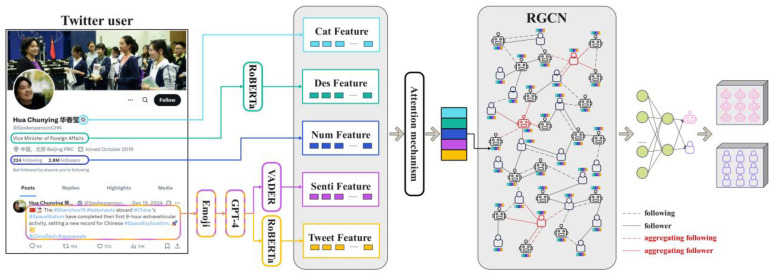
ESA-BotRGCN model diagram.

According to the aforementioned discussion, existing social bot detection methods predominantly suffer from two major limitations. First, the rich semantic and emotional expressive value of emojis is often overlooked, as most studies treat them as noise and directly remove them during preprocessing, leading to the loss of critical features [11]. Second, there exist significant statistical deviations in emotional expression patterns between bot accounts and genuine users, including differences in emotional complexity, polarity span, dynamic fluctuations, and expression consistency. However, these discriminative features have yet to be fully explored [42]. To address these issues, this study innovatively proposes ESA-BotRGCN, a Graph Convolutional Network model enhanced by emoji-driven sentiment and semantics, which dynamically integrates multi-dimensional features to improve the accuracy and robustness of social bot detection. Figure 3 illustrates the architecture of our proposed model.

The detection method proposed in this study achieves end-to-end modeling through multi-stage collaborative processing. First, tweets undergo preprocessing; emojis are converted into corresponding textual descriptions using the Emoji Library. The GPT-4 model is then employed to refine the textual coherence of the tweets. Next, the RoBERTa model is utilized to generate tweet embeddings that integrate emoji semantics, thereby addressing the limitations of traditional word vectors in representing the semantic information conveyed by emojis and enhancing the expressiveness of sentiment features. Subsequently, seven sentiment-based features are extracted from the preprocessed tweets to capture statistical differences in emotional expression patterns between bot and human accounts. ESA-BotRGCN then jointly encodes tweet features, sentiment-based features, user description features, numerical attributes, and categorical features. Through an attention gating mechanism, the model dynamically adjusts the weights of these heterogeneous features to generate a globally context-aware unified feature vector. This comprehensive feature vector is fed into an RGCN, where two layers of graph convolution operations integrate the topological structure of user social relationships, and dropout is applied to suppress overfitting. Finally, the output features are mapped to a high-dimensional space via a linear layer, and the detection probability of bot accounts is computed using the Softmax function. The Adam optimizer is employed to perform gradient backpropagation based on the cross-entropy loss function, optimizing model parameters to obtain the final classification results.

### 3.1. Emoji Preprocessing

According to the aforementioned discussion, existing social bot detection studies often treat emojis as noise and directly remove them during preprocessing [11], resulting in severe loss of semantic and emotional cues in tweets. Particularly in short tweets, a single emoji can significantly alter the meaning of the entire tweet. To address this issue, this study employs emoji-to-text mapping and language model optimization to fully preserve the semantic and emotional value of emojis. First, each tweet t is defined as an ordered joint structure of textual tokens and emojis:

(1) t={w,e}

In this formula, $w=\{w_{1},w_{2},\ldots ,w_{M}\}$ denotes the ordered sequence of textual tokens in the tweet, with M being the total number of words and $e=\{e_{1},e_{2},\ldots ,e_{K}\}$ denoting the ordered sequence of emojis in the tweet, where K is the total number of emojis. The preprocessing workflow includes the following steps:

#### 3.1.1. Emoji Mapping

First, emoji detection is performed on the tweet. If no emojis are detected, no further processing is applied. If emojis are present, each emoji $e_{k}$ is converted into its corresponding textual description ${\mathrm{text}}_{k}$ using a predefined mapping table M:

(2) $$f(e_{k})={\mathrm{text}}_{k}\;(k=1,2,\ldots ,K)$$

This study compared the Unicode coverage, multilingual description support, semantic mapping accuracy, and update frequency and found that Emoji Library outperformed both Emojiswitch Library and EmojiBase Library: it fully covers the entire Unicode 15.0 version, including the emojis added in 2025, whereas Emojiswitch only covers up to Unicode 13.0 and EmojiBase covers up to 14.0 with some missing variants. Additionally, Emoji Library provides long-text descriptions in multiple languages, such as English and Chinese, while the other two only support English descriptions. Finally, Emoji Library maintains quarterly updates to keep pace with official Unicode releases, which is more adaptable to the semantic evolution of emojis compared to Emojiswitch’s annual updates and EmojiBase’s irregular updates. Therefore, this study selected the Emoji Library to construct the emoji–text mapping table M. As shown in Table 1, which lists 10 typical mapping examples.

The mapped tweet $t^{\prime }$ is expressed as follows:

(3) $$t^{\prime }=w\cup \{f(e_{1}),f(e_{2}),\ldots ,f(e_{K})\}$$

#### 3.1.2. Text Coherence Optimization

**Figure 4:**
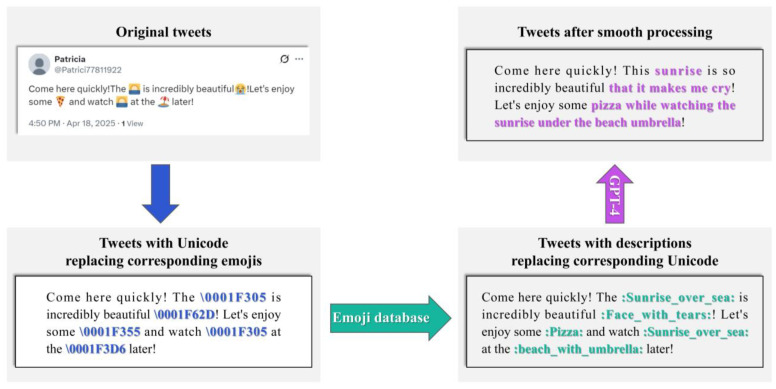
Tweet processing flow: from emojis to Unicode and descriptions using GPT-4.

Tweets converted from emojis often suffer from poor textual coherence and semantic ambiguity. For example, the tweet “Let’s go to the beach! 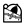
” becomes “Let’s go to the beach! :beach_with_umbrella:” after conversion, resulting in semantic discontinuity. To address this, this study introduces the GPT-4 model for semantic reconstruction; the prompt is: “You are a language analysis expert. Analyze the tweet provided to you. The emojis in the tweet have been converted into corresponding text label embeddings. Please make the tweet coherent without changing its original meaning, and ensure the length of the generated tweet does not exceed 200% of the original tweet.”. As illustrated in Figure 4, this process ensures that tweets fully express their semantic and emotional information. For instance, the aforementioned tweet is reconstructed as “Let’s go to the beach and enjoy the sun under an umbrella!”.

#### 3.1.3. Handling of Abnormal Emojis

For rare or custom emojis not included in the Emoji Library, this study retains their original Unicode encoding and appends the special token “[UNK]” as a placeholder to avoid information loss. Subsequently, all modified tweets are fed into the model for training instead of the original tweets.

### 3.2. Sentiment-Based Features Processing

Sentiment-based features $r_{p}^{\mathrm{senti}}$ capture statistical differences in emotional expression patterns between bot and human accounts by quantifying the sentiment dynamics in user tweets. The innovation of this study lies in proposing seven sentiment features, including four foundational ones—positive, negative, and neutral sentiment values, as well as sentiment complexity—and three advanced features derived from these, including sentiment polarity span, volatility, and consistency. These features significantly enhance discriminative power. The specific definitions are as follows:

#### 3.2.1. Basic Sentiments and Complexity

This study employs VADER [43] for analyzing basic sentiment values. The selection of VADER is attributed to three advantages it possesses. First, VADER is adaptable to the characteristics of social media texts. It can effectively handle fragmented content such as short texts and informal language (e.g., slang) in tweets. Its built-in sentiment dictionary contains a large number of commonly used social media words (e.g., “OMG”). Second, VADER does not require a large amount of labeled data. It is lightweight and efficient, enabling sentiment analysis solely through predefined sentiment dictionaries and grammatical rules. It has low computational costs and is easy to deploy, making it suitable for processing large-scale tweet data. Third, VADER can calculate sentiment scores by decomposing elements such as sentiment words, negation words, and intensifier words in the text. The results have clear interpretability, facilitating the analysis of differences in sentiment expression between bots and real users. In recent years, VADER has seen many typical and effective application cases. For example, Francesco et al. [44] applied VADER to investigate the sentiment experiences among e-sports spectators, and Anny et al. [45] used VADER to classify sentiments and discovered the correlation between Twitter sentiments and the price movements of Bitcoin. VADER is highly compatible with the multimodal sentiment feature extraction requirements of this study. Therefore, VADER is adopted to analyze basic sentiment values, and the specific process is as follows:

Conduct sentiment analysis on each tweet of the user. Based on a predefined sentiment dictionary, which contains the positive, negative, and neutral sentiment intensity values for each word, combined with context-adjustment rules (such as negative words, emphasis words, punctuation marks, etc.), extract the positive sentiment value ($S_{\mathrm{Pos}}^{\left(i\right)}$), negative sentiment value ($S_{\mathrm{Neg}}^{\left(i\right)}$), neutral sentiment value ($S_{\mathrm{Neu}}^{\left(i\right)}$), and sentiment complexity ($S_{\mathrm{Com}}^{\left(i\right)}$):

(4) $$S_{Pos}^{\left(i\right)}=\sum_{j=1}^{M}I_{Pos}(w_{j})\cdot C(w_{j})$$

(5) $$S_{Neg}^{\left(i\right)}=\sum_{j=1}^{M}I_{Neg}(w_{j})\cdot C(w_{j})$$

In this formula, i denotes the i-th tweet, j represents the j-th word in the i-th tweet, and M is the total number of words in the i-th tweet. $I_{Pos}(w_{j})$ and $I_{Neg}(w_{j})$, respectively, denote the positive and negative sentiment intensities of each word $w_{j}$, and $C(w_{j})$ represents the contextual adjustment factor.

(6) $$S_{Neu}^{\left(i\right)}=1-(S_{Pos}^{\left(i\right)}+S_{Neg}^{\left(i\right)})$$

Given that both $S_{\mathrm{Pos}}^{\left(i\right)}+S_{\mathrm{Neg}}^{\left(i\right)}+S_{\mathrm{Neu}}^{\left(i\right)}=1$ and $S_{\mathrm{Pos}}^{\left(i\right)}+S_{\mathrm{Neg}}^{\left(i\right)}\neq 1$ always hold, the neutral sentiment value corresponds to the residual portion not covered by positive or negative sentiments.

Sentiment complexity $S_{\mathrm{Com}}^{\left(i\right)}$, which quantifies the diversity and volatility of emotional expression, is measured via entropy or standard deviation as follows:

(7) $$S_{Com}^{\left(i\right)}=-\sum_{c\in \{Pos,Neg,Neu\}}P_{c}\cdot \log(P_{c})$$

In this formula, $P_{c}=\frac{S_{c}^{\left(i\right)}}{S_{Pos}^{\left(i\right)}+S_{Neg}^{\left(i\right)}+S_{Neu}^{\left(i\right)}}$ is the normalized proportion of each sentiment category. Finally, the mean of sentiment features across all tweets from users is computed to derive the user-level sentiment feature vector:

(8) $${\bar{S}}_{\mathrm{Pos}}=\frac{1}{N}\sum_{i=1}^{N}S_{\mathrm{Pos}}^{\left(i\right)}$$

(9) $${\bar{S}}_{\mathrm{Neg}}=\frac{1}{N}\sum_{i=1}^{N}S_{\mathrm{Neg}}^{\left(i\right)}$$

(10) $${\bar{S}}_{\mathrm{Neu}}=\frac{1}{N}\sum_{i=1}^{N}S_{\mathrm{Neu}}^{\left(i\right)}$$

(11) $${\bar{S}}_{\mathrm{Com}}=\frac{1}{N}\sum_{i=1}^{N}S_{\mathrm{Com}}^{\left(i\right)}$$

In this formula, i denotes the i-th tweet; N is the total number of tweets by the user; ${\bar{S}}_{\mathrm{Pos}}$ and ${\bar{S}}_{\mathrm{Neg}}$ reflect the overall positive and negative emotional tendencies of the user, respectively; ${\bar{S}}_{\mathrm{Neu}}$ characterizes the degree of emotional neutrality; and ${\bar{S}}_{\mathrm{Com}}$ measures the complexity of emotional expression.

#### 3.2.2. Sentiment Polarity Span

To capture the range of sentiment fluctuations in sentiment expressions, the sentiment polarity span $S_{\mathrm{Span}}$ is defined as the absolute difference between the maximum positive sentiment value and the maximum negative sentiment value across all tweets from the same user:

(12) $$S_{\mathrm{Span}}=\max_{1\leq i\leq N}S_{\mathrm{Pos}}^{\left(i\right)}+\max_{1\leq i\leq N}S_{\mathrm{Neg}}^{\left(i\right)}$$

Here, $S_{\mathrm{Pos}}^{\left(i\right)}$ and $S_{\mathrm{Neg}}^{\left(i\right)}$ are both positive values. This metric quantifies the extremity of a user’s emotional expression. Real users, due to the diversity of life events (such as celebrating victories and encountering setbacks), will have significant positive and negative emotional fluctuations, and $S_{\mathrm{Span}}$ is usually relatively high; however, robots, restricted by task objectives (such as conducting concentrated propaganda or attacks), have their emotional expressions constrained within a specific polarity range, and $S_{\mathrm{Span}}$ is significantly low. Even if attackers attempt to simulate the human emotional range, it will reduce the attack efficiency due to disrupting task consistency. Therefore, this feature can significantly improve the performance of robot detection.

#### 3.2.3. Sentiment Volatility

To further quantify the volatility of emotional expression, sentiment volatility $S_{\mathrm{Var}}$ is introduced, measured as the standard deviation of sentiment complexity across a user’s tweets:

(13) $$S_{\mathrm{Var}}=\sqrt{\frac{1}{N-1}\sum_{i=1}^{N}{\left(S_{\mathrm{Com}}^{\left(i\right)}-{\bar{S}}_{\mathrm{Com}}\right)}^{2}}$$

Sentiment volatility reveals the differences in randomness during the evolution of emotions. Human emotions, triggered by immediate events, exhibit irregular fluctuations (such as a shift from anger to surprise caused by breaking news). Thus, the sentiment complexity of real users shows relatively large fluctuations ($S_{\mathrm{Var}}$ is high). In contrast, robots generate content in a programmatic manner, so the distribution of their sentiment complexity is more concentrated ($S_{\mathrm{Var}}$ is low). If robots forcibly inject random fluctuations to evade detection, it will lead to a decline in semantic coherence and expose traces of generation.

#### 3.2.4. Sentiment Consistency

Sentiment consistency $S_{\mathrm{Consist}}$ is defined as the mean cosine similarity of sentiment complexity between adjacent tweets, capturing the continuity of a user’s emotional expression:

(14) $$S_{\mathrm{Consist}}=\frac{1}{N-1}\sum_{i=1}^{N-1}\frac{v_{i}\cdot v_{i+1}}{\left\|v_{i}\|v_{i+1}\right\|}$$

In this formula, $v_{i}=[S_{\mathrm{Pos}}^{\left(i\right)},S_{\mathrm{Neg}}^{\left(i\right)},S_{\mathrm{Com}}^{\left(i\right)}]$ represents the sentiment vector of the tweet. Robot accounts generate purposeful content in batches. For example, sending 50 consecutive advertising tweets will lead to a high degree of similarity in sentiment between adjacent tweets ($S_{\mathrm{Consist}}$ approaches 1). In contrast, human beings switch topics more frequently than robots (such as shifting from sports discussions to food sharing), and their sentiment expressions are more random, resulting in low sentiment continuity ($S_{\mathrm{Consist}}$ is relatively low). If a robot deliberately reduces consistency, it needs to sacrifice task focus, significantly weakening its propagation effect.

The sentiment polarity span, sentiment volatility, and sentiment consistency form a triangular constraint on sentiment expression, bringing about a joint defense advantage. If a robot attempts to adjust one feature (such as increasing the polarity span to simulate human beings), it will simultaneously disrupt other features (such as causing an abnormal increase in volatility or a break in consistency), forming an “impossible triangle” for detection evasion.

Finally, the sentiment feature vector is constructed by concatenating the above metrics:

(15) $$r_{p}^{\mathrm{senti}}=[{\bar{S}}_{\mathrm{Pos}},{\bar{S}}_{\mathrm{Neg}},{\bar{S}}_{\mathrm{Neu}},{\bar{S}}_{\mathrm{Com}},S_{\mathrm{Span}},S_{\mathrm{Var}},S_{\mathrm{Consist}}]\in {\mathbb{R}}^{7}$$

### 3.3. User Node Feature Processing

ESA-BotRGCN aims to leverage multi-modal user information to address the challenge of bot account camouflage, enabling accurate identification of malicious bot accounts and preventing the spread of misinformation. Specifically, ESA-BotRGCN jointly encodes five categories of features: sentiment-based features, user description features, tweet content features, numerical attributes, and categorical features. In this section, the encoding process of the latter four types of features will be elaborated in detail.

#### 3.3.1. User Description Feature $r_{b}$

User description features are extracted from the personal bio on the account homepage. To capture their semantic information, a pre-trained RoBERTa model is employed for encoding. First, the user description text is input into RoBERTa to generate word-level embedding vectors:

(16) $$\bar{b}=RoBERTa({\left\{b_{i}\right\}}_{i=1}^{L}),\;\bar{b}\in {\mathbb{R}}^{D_{s}\times 1}$$

In this formula, $\bar{b}$ represents the user description embedding, i denotes the i-th word in the description, L is the total number of words, and $D_{s}$ is the embedding dimension of RoBERTa. The user description representation vector is then derived as follows:

(17) $$r_{b}=\phi (W_{B}\cdot \bar{b}+b_{B}),\;r_{b}\in {\mathbb{R}}^{D/4\times 1}$$

In this formula, $W_{B}$ and $b_{B}$ are learnable parameters, ϕ is the activation function, and D is the embedding dimension for Twitter users. In subsequent sections of this paper, Leaky-ReLU is adopted as ϕ.

#### 3.3.2. Tweet Content Feature $r_{t}$

After emoji preprocessing (Section 3.1), the semantic and emotional information of tweets is enhanced. The processed tweets are encoded using RoBERTa in a manner similar to the above. First, RoBERTa generates an embedding vector $t_{i}\in {\mathbb{R}}^{D}$ for each tweet. The mean of all tweet embeddings for a user is then computed to obtain the user-level tweet representation:

(18) $$r_{t}=\frac{1}{N}\sum_{i=1}^{N}t_{i}$$

In this formula, N is the total number of tweets by the user.

#### 3.3.3. Numerical Features $r_{p}^{num}$

Numerical attributes refer to measurable metrics that quantify user behaviors and attributes, as listed in Table 2. These features provide numerical descriptions of user behavioral patterns and preferences. The numerical attributes are processed using Multi-Layer Perceptrons (MLPs) and graph neural networks (GNNs). Specifically, 10 numerical features are first directly retrieved from the Twitter API, with the first three being emoji-related. Z-score normalization is then applied to eliminate scale differences, followed by a fully connected layer to derive the user’s numerical feature representation $r_{p}^{num}$.

#### 3.3.4. Categorical Features $r_{p}^{cat}$

Categorical features are qualitative indicators describing user attributes, as shown in Table 3. These features are typically represented in binary form (yes/no). Similar to numerical attributes, we avoid manual feature engineering and instead encode them using MLPs and GNNs. Specifically, 11 categorical features are directly retrieved from the Twitter API. One-hot encoding is applied to convert these features into sparse vectors, which are then concatenated and transformed via a fully connected layer with Leaky-ReLU activation, yielding the user categorical feature representation $r_{p}^{cat}$.

### 3.4. Features Fusion

The proposed model achieves multi-modal feature fusion through feature encoding and an attention mechanism. Given a set of user node features $\left\{r_{p}^{\mathrm{senti}},r_{b},r_{t},r_{p}^{num},r_{p}^{cat}\right\}$, representing sentiment-based features, user descriptions, tweet content, numerical attributes, and categorical features, respectively; the fusion process is defined as follows:

#### 3.4.1. Feature Alignment and Nonlinear Transformation

Heterogeneous features are projected into a unified dimensional space via fully connected layers to eliminate dimensional discrepancies and introduce nonlinear expressive capabilities:

(19) $${\tilde{r}}_{k}=\mathrm{LeakyReLU}\left(W_{k}r_{k}+b_{k}\right),\;k\in \{\mathrm{senti},b,i,num,cat\}$$

In this formula, $W_{k}\in {\mathbb{R}}^{D\times d_{k}}$ is the learnable weight matrix, $b_{k}\in {\mathbb{R}}^{D}$ is the bias term, D = 256 is the unified embedding dimension, and Leaky-ReLU mitigates gradient vanishing.

#### 3.4.2. Single-Head Attention Weight Allocation

A single-head attention mechanism dynamically evaluates feature importance through query–key interactions:

(20) $$\alpha_{k}=\frac{\exp\left(q^{T}(W_{a}{\tilde{r}}_{k})\right)}{\sum_{j\in \{senti,b,i,num,cat\}}\exp\left(q^{T}(W_{a}{\tilde{r}}_{j})\right)}$$

In this formula, $W_{a}\in {\mathbb{R}}^{D\times D}$ is a parameter matrix and $q\in {\mathbb{R}}^{D}$ is a learnable global query vector that captures cross-feature global dependencies.

#### 3.4.3. Context-Aware Feature Fusion

A globally integrated feature vector $r_{i}\in {\mathbb{R}}^{D}$ is generated via weighted summation and fed into the subsequent RGCN module for social graph modeling:

(21) $$r_{i}=\sum_{k\in \{senti,b,i,num,cat\}}\alpha_{k}{\tilde{r}}_{k}$$

Through this approach, the model dynamically adjusts the influence of each feature during training, thereby enhancing the discernment of feature importance. This fusion method enables the model to effectively integrate information from diverse sources and deliver superior performance in complex tasks.

### 3.5. Heterogeneous Social Graph Modeling

To address the limitations of traditional graph neural networks in modeling multi-relational data, this study employs RGCN [46] for deep feature extraction from heterogeneous social graphs. We construct a heterogeneous information network G=(V,E,R), where the node set V represents user accounts, the edge set E includes two relationship types $R=\{r_{1},r_{2}\}=\{“following”,“follower”\}$, characterizing active and passive follow behaviors between users. Each user node $v_{i}\in V$ is associated with a feature vector $r_{i}$, derived from the attention-based fusion in Section 3.3. RGCN integrates rich contextual information from nodes through efficient message passing and aggregation mechanisms. During this process, RGCN not only considers the intrinsic features $r_{i}$ of node $v_{i}$ but also incorporates neighborhood information to generate context-aware node representations. This multi-layer stacking progressively expands the receptive field, capturing global topological patterns. Furthermore, social networks are typically large-scale and sparse, with limited node connections, posing challenges for information propagation. RGCN overcomes these limitations via a dynamic relation-aware edge weighting mechanism, which updates relationship weights and relation-specific embeddings to address potential information gaps in the graph. The graph convolution operations of RGCN are implemented as follows.

#### 3.5.1. Node Initialization

The user feature vector $r_{i}$ undergoes linear transformation and activation function to generate the initial hidden representation:

(22) $$x_{i}^{\left(0\right)}=\phi (W_{1}r_{i}+b_{1}),\;x_{i}^{\left(0\right)}\in {\mathbb{R}}^{D\times 1}$$

In this formula, $W_{1}$ is a learnable weight matrix, $b_{1}$ is a bias term, and ϕ is the Leaky-ReLU activation function.

#### 3.5.2. Relational Graph Convolution

At the l-th layer, the representation of node $v_{i}$ is updated as follows:

(23) $$x_{i}^{\left(l+1\right)}=\Theta_{\mathrm{self}}x_{i}^{\left(l\right)}+\sum_{r\in R}\sum_{j\in N_{r}(i)}\frac{1}{\left|N_{r}(i)\right|}\Theta_{r}x_{j}^{\left(l\right)},x_{i}^{\left(l+1\right)}\in {\mathbb{R}}^{D\times 1}$$

Here, $N_{r}(i)$ denotes the set of neighbors of $v_{i}$ under relation r, and $\Theta_{\mathrm{self}}$ and $\Theta_{r}$ are the weight matrices for self-connections and relationship r, respectively.

#### 3.5.3. Multi-Layer Perceptron (MLP) Enhancement

Stacked RGCN layers are interleaved with MLPs to further refine node representations and expand the receptive field:

(24) $$h_{i}={\mathrm{MLP}}^{\left(l\right)}\left(x_{i}^{\left(l\right)}\right)=\phi (W_{\mathrm{MLP}}^{\left(l\right)}x_{i}^{\left(l\right)}+b_{\mathrm{MLP}}^{\left(l\right)})$$

In this formula, $W_{\mathrm{MLP}}^{\left(l\right)}$ and $b_{\mathrm{MLP}}^{\left(l\right)}$ are learnable parameters. The final node representation $h_{i}$ is mapped to a high-dimensional discriminative space via the MLP.

### 3.6. Learning and Optimization

ESA-BotRGCN formulates social bot detection as a binary classification task, where the label $y_{i}\in \{0,1\}$ of a user account denotes a genuine user (0) or a social bot (1). This section details the classification mechanism and loss function design.

#### 3.6.1. Classifier Design

The model leverages context-aware node representations $h_{i}\in {\mathbb{R}}^{128}$, extracted by the RGCN, to construct the classifier. First, the node representation $h_{i}$ is mapped to a 2D space via a fully connected layer:

(25) $$z_{i}=W_{O}\cdot h_{i}+b_{O}$$

In this formula, $W_{O}\in {\mathbb{R}}^{128\times 2}$ is the weight matrix and $b_{O}\in {\mathbb{R}}^{2}$ is the bias term. The class probability distribution is then computed using the Softmax function:

(26) $${\hat{y}}_{i}=\mathrm{Softmax}(z_{i})=\left[\frac{e^{z_{i0}}}{e^{z_{i0}}+e^{z_{i1}}},\frac{e^{z_{i1}}}{e^{z_{i0}}+e^{z_{i1}}}\right]$$

#### 3.6.2. Loss Function

The total loss combines cross-entropy loss and a weight decay term to balance classification accuracy and model complexity:

(27) $$L=-\sum_{i\in Y}\left[y_{i}\log({\hat{y}}_{i})+(1-y_{i})\log(1-{\hat{y}}_{i})\right]+\lambda \sum_{w\in \theta }w^{2}$$

The first part is the cross-entropy loss, which measures the deviation between the predicted probability and the true label to evaluate the accuracy of the prediction. The second part is the weight decay term. $\sum_{i\in y}$ represents the summation over all labeled users, $\sum_{w\in \theta }$ represents the summation over all learnable parameters in the ESA-BotRGCN framework, and λ is the decay coefficient used to control the model parameters and suppress overfitting.

## 4. Experiments

### 4.1. Experimental Setup

#### 4.1.1. Dataset

This study uses the publicly available Twitter bot detection benchmark datasets TwiBot-20 [47] and Cresci-15 [48] to conduct experimental verification.

The TwiBot-20 dataset contains 229,580 user nodes, 33,488,192 tweet texts, 8,723,736 user attribute data entries, and 455,958 bidirectional follow relationships. Following the original data partitioning strategy, we employed a stratified sampling method to divide the dataset into training (80%), validation (10%), and test (10%) sets, ensuring balanced class distribution. Furthermore, based on user nodes and follow relationships, we constructed a heterogeneous graph structure where nodes represent user entities and edges represent unidirectional follow behaviors, ultimately generating a social network topology comprising 229,580 nodes and 227,979 directed edges.

The Cresci-15 dataset consists of five sub-datasets, with a total of 5301 users and 7,086,134 edges. All accounts are labeled, among which 3351 accounts are marked as bot accounts. Similar to the TwiBot-20 dataset, we divide it into a training set (80%), a validation set (10%), and a test set (10%).

#### 4.1.2. Evaluation Metrics

To comprehensively evaluate model performance, this study adopts the following three metrics.

1. Accuracy: Measures the overall proportion of correct classifications.

(28) $$\mathrm{Accuracy}=\frac{\mathrm{TP}+\mathrm{TN}}{\mathrm{TP}+\mathrm{TN}+\mathrm{FP}+\mathrm{FN}}$$

In this formula, TP (true positive) denotes the number of correctly identified bot accounts, TN (true negative) represents the number of correctly identified genuine users, and FP (false positive) and FN (false negative) indicate the numbers of misclassified positive and negative samples, respectively.

2. F1-score [49]: The harmonic mean of precision and recall, suitable for scenarios requiring balanced class distributions.

(29) $$F1\text{-}\mathrm{score}=2\times \frac{\mathrm{Precision}\times \mathrm{Recall}}{\mathrm{Precision}+\mathrm{Recall}}$$

In this formula, the meanings of precision and recall are as follows:

(30) $$\mathrm{Precision}=\frac{\mathrm{TP}}{\mathrm{TP}+\mathrm{FP}}$$

(31) $$\mathrm{Recall}=\frac{\mathrm{TP}}{\mathrm{TP}+\mathrm{FN}}$$

3. MCC (Matthews Correlation Coefficient) [50]: A statistical measure that synthesizes all four elements of the confusion matrix, robust to class imbalance.

(32) $$\mathrm{MCC}=\frac{\mathrm{TP}\times \mathrm{TN}-\mathrm{FP}\times \mathrm{FN}}{\sqrt{\left(\mathrm{TP}+\mathrm{FP})(\mathrm{TP}+\mathrm{FN})(\mathrm{TN}+\mathrm{FP})(\mathrm{TN}+\mathrm{FN}\right)}}$$

MCC’s range is [−1, 1], where a value closer to 1 indicates superior model performance.

The aforementioned metrics evaluate the model’s classification capabilities from different dimensions. Accuracy reflects overall correctness, the F1-score balances class sensitivity, and MCC mitigates the impact of class distribution skew. Their combination can comprehensively validate the robustness of the detection framework.

#### 4.1.3. Baseline Methods

We selected 10 mainstream social bot detection methods as baseline models for performance comparison with ESA-BotRGCN, covering diverse technical approaches including traditional machine learning, deep learning, and graph neural networks. The specific methods are as follows.

- Lee et al. [51]: A Random Forest-based model that integrates multi-dimensional features such as user-following networks and tweet content for detection.
- Yang et al. [52]: Employs lightweight metadata combined with a Random Forest algorithm to achieve efficient tweet stream analysis.
- Kudugunta et al. [53]: Leverages a contextual Long Short-Term Memory (LSTM) network to jointly model tweet content and metadata for tweet-level bot detection.
- Wei et al. [54]: Utilizes a three-layer Bidirectional Long Short-Term Memory (BiLSTM) with word embeddings to identify social bots on Twitter.
- Miller et al. [55]: Treats bot detection as an anomaly detection problem rather than classification, introducing 95 lexical features extracted from tweet text.
- Cresci et al. [26]: Innovatively encodes user behaviors into DNA-like sequences and distinguishes bots from humans via Longest Common Subsequence (LCS) similarity.
- Botometer [7]: A widely used public Twitter detection tool that employs over 1000 features.
- Alhosseini et al. [56]: Detects social bots using a Graph Convolutional Neural Network (GCNN) by leveraging node features and aggregated neighborhood node features.
- SATAR [57]: A self-supervised representation learning framework capable of generalizing by jointly utilizing user semantics, attributes, and neighborhood information.
- BotRGCN [36]: Addresses bot detection by constructing a heterogeneous graph from Twitter user-follow relationships and applying an RGCN.

### 4.2. Comparative Experiments

This section validates the effectiveness of each module in the ESA-BotRGCN model through four sets of ablation experiments, including the impact of emoji preprocessing, sentiment feature integration, the attention mechanism, and the selection of graph neural network architecture and its layer count on detection performance. Additionally, we have conducted statistical significance analysis under different feature settings.

#### 4.2.1. Emoji Preprocessing Ablation Study

To validate the importance of emojis in tweet semantics and sentiment analysis, this section compares the impact of four tweet input formats on detection performance under the framework of using seven-dimensional advanced sentiment features and a two-layer RGCN-based graph neural network architecture.

- Original tweets: Raw tweets without any emoji processing.
- Emoji-mapped tweets: Tweets where emojis are replaced with corresponding textual descriptions from the Emoji Library.
- GPT-4 optimized tweets: Emoji-mapped tweets further refined by GPT-4 for textual coherence.
- Qwen-2.5 Optimizes Tweets: Use Qwen-2.5, owned by Alibaba, to optimize the text coherence of tweets after mapping with the Emoji library, and keep the prompt consistent with that of GPT-4.

As shown in Table 4, the original tweets yielded the lowest detection performance due to the neglect of emoji semantics and emotional cues. Mapping emojis to textual descriptions via the Emoji Library enabled the model to capture semantic and emotional associations, significantly improving performance. Tweets optimized by large language models (LLMs) further enhance text coherence, and GPT-4, in particular, achieves even better results. This enables the model to more accurately extract semantic and sentiment features, thereby attaining optimal performance. Experimental results demonstrate that semantic reconstruction and coherence optimization of emojis play a critical role in improving detection accuracy.

Notably, while LLMs improve text coherence and enhance detection accuracy, their computational cost should be taken into account: the inference time for 10,000 tweets on an A100 GPU is 2.1 h (Qwen-2.5) and 2.6 h (GPT-4). This trade-off between performance and cost makes LLMs suitable for scenarios where detection accuracy is prioritized over real-time deployment.

#### 4.2.2. Sentiment Feature Ablation Study

To validate the contribution of sentiment features, this section conducts ablation experiments under three configurations, each employing preprocessed tweets and a two-layer RGCN-based graph neural network architecture.

- No sentiment features: Only user descriptions, tweet content, numerical attributes, and categorical features are used.
- Basic sentiment features: Incorporates positive, negative, and neutral sentiment polarities, as well as sentiment complexity.
- Advanced sentiment features: On the basis of basic sentiment features, three additional features are added—polarity span, volatility, and consistency.

As shown in Table 5, the model performance significantly declined when no sentiment features were used, indicating that sentiment features are critical clues for distinguishing bots from genuine users. After introducing four basic sentiment features, accuracy was partially improved, and further incorporating polarity span, volatility, and consistency into the seven-dimensional sentiment features significantly enhanced the model’s ability to capture emotional expression patterns, ultimately achieving an accuracy of 87.46%. The experiments demonstrate that these seven sentiment features can effectively reflect the differences in emotional expression between bot accounts and genuine users, thereby significantly enhancing the model’s discriminative capability.

#### 4.2.3. Selection of Graph Neural Network Architecture and Its Layers

To validate the advantages of RGCN in modeling heterogeneous social graphs, this study compares the detection performance of different graph neural network architectures, each employing preprocessed tweets and seven-dimensional advanced sentiment features.

- Graph Attention Network (GAT): Dynamically assigns edge weights through an attention mechanism.
- Graph Convolutional Network (GCN): Models homogeneous social relationships without distinguishing node or edge types.
- Fully Connected Neural Network: Applies nonlinear transformations via Multi-Layer Perceptrons.

As shown in Table 6, while GAT introduces an attention mechanism, its stability in modeling complex relationships remains insufficient. The GCN model ignores relational heterogeneity, failing to differentiate between active following and passive following behaviors, resulting in suboptimal performance. The fully connected neural network achieves better classification performance through effective feature learning and processing. Although the neural network achieves high detection accuracy through feature fusion, it essentially belongs to a node-level feature processing model and cannot capture the implicit topological structure information in social networks. With the increase in social interaction density, the connections between users are becoming closer and closer, and the dynamic changes in network topology put forward higher requirements for the model. The multi-layer message-passing mechanism of RGCN can gradually aggregate multi-order neighbor information and maintain the stable extraction of topological features in dense networks, which elevates the social bot detection from “individual behavior recognition” to the level of “group structure understanding”. In the experiment, RGCN achieved optimal performance, thus demonstrating its indispensability.

**Figure 5:**
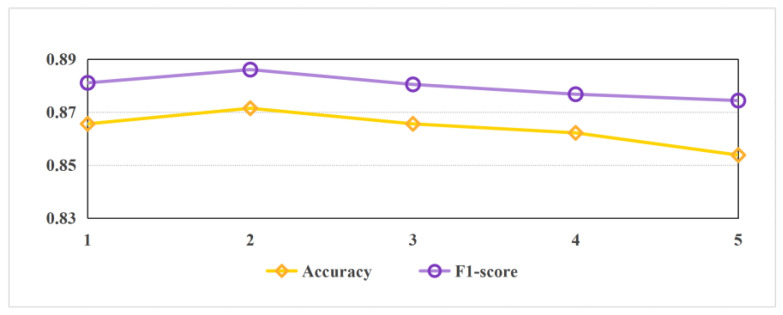
Performance comparison of RGCN with varying layer numbers.

Furthermore, to further investigate the impact of RGCN layer depth on model performance, this study conducted comparative experiments on RGCN architectures with varying numbers of layers. As shown in Figure 5, the 1-layer RGCN failed to adequately aggregate global topological information due to its limited receptive field, while models with three or more layers suffered from performance degradation caused by over-smoothing. Ultimately, the 2-layer RGCN achieved an optimal balance between parameter efficiency and feature representation capability, validating the rationality of the current architectural design.

#### 4.2.4. Ablation Experiment on Attention Mechanism

To verify the effectiveness of the attention gating mechanism in multi-modal feature fusion, this section designs the following two sets of comparative experiments while keeping other variables unchanged (that is, all use preprocessed tweets, seven-dimensional advanced emotional features, and a two-layer RGCN architecture).

- Without-attention mechanism: Directly concatenate five types of feature vectors (sentiment features, user descriptions, tweet content, numerical features, and categorical features) to replace attention-weighted fusion.
- Complete-attention mechanism: Use the attention gating network proposed in Section 3.4 of this paper for dynamic feature fusion.

The experimental results are shown in Table 7. After removing the attention mechanism, the performance of ESA-BotRGCN declines comprehensively, indicating that simple feature concatenation is difficult to effectively distinguish feature importance. The attention mechanism dynamically allocates weights, enabling ESA-BotRGCN to dynamically adjust the influence of various features during the training process, thereby improving the ability to recognize feature importance. This fusion method allows the model to more effectively integrate information from different sources and provide better performance in complex tasks.

#### 4.2.5. Statistical Significance Analysis of Different Feature Settings

To further validate the independent and joint contributions of emotional features and emoji preprocessing to performance enhancement, this section compares the performance of three model variants against the complete ESA-BotRGCN model and applies the Welch’s *t*-test to assess whether the observed differences are statistically significant. Specifically, the following three simplified model settings were used for comparison:

- Without emotion and emojis: sentiment features and emoji preprocessing were removed, retaining only the other feature modalities.
- Use only sentiment features: Retain the seven-dimensional sentiment features and remove the text enhancement processing for emojis.
- Use only emojis: Perform emoji-to-text mapping and GPT-4 semantic optimization, but do not introduce sentiment features.

As shown in Table 8, the average performance of all three simplified variants differs significantly from that of the complete model. The “without emotion and emojis” version exhibited the largest performance gap (t = −16.82, *p* < 0.00001), indicating an extremely significant difference and highlighting the importance of integrating both sentiment and emoji-related features. In addition, introducing either sentiment features alone (t = −9.25, *p* = 0.000099) or emoji preprocessing alone (t = −8.09, *p* = 0.000045) also resulted in statistically significant improvements, thereby confirming that both information sources contribute independently to model performance.

It is worth noting that the t-value in a Welch’s *t*-test measures the standardized difference between the means of two groups relative to their variance. A negative t-value indicates that the complete model outperforms the comparison version. The corresponding *p*-value represents the probability of observing such a result under the null hypothesis (i.e., no significant difference). When the *p*-value is substantially lower than the commonly accepted threshold (e.g., 0.05), the likelihood that the observed effect is due to random variation is minimal, thus providing strong statistical evidence to reject the null hypothesis and support the alternative—that the proposed feature processing yields a positive and significant impact on model performance.

### 4.3. Performance Comparison

**Figure 6:**
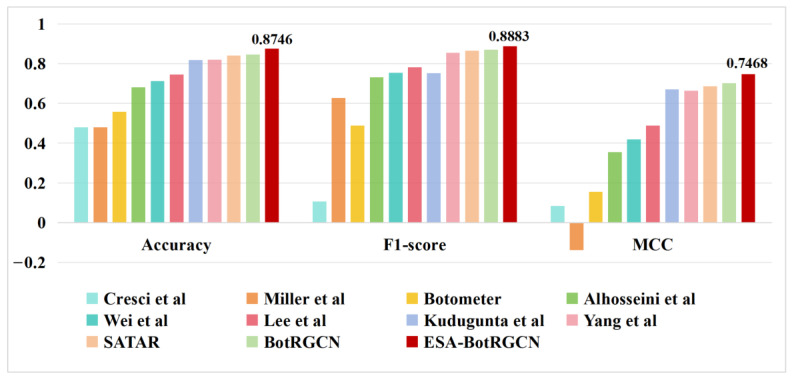
Comparison of classification performance metrics for various models on TwiBot-20 [27,51,52,53,54,55,56].

Table 9 shows the social bot detection performance of ESA-BotRGCN compared with the baseline models from Section 4.1.3 on the TwiBot-20 dataset. Table 10 shows the social bot detection performance of ESA-BotRGCN and seven mainstream baseline models on the Cresci-15 dataset. The results in Figure 6 demonstrate that ESA-BotRGCN significantly outperforms all other methods on TwiBot-20, confirming its general effectiveness in Twitter social bot detection tasks. Additionally, ESA-BotRGCN achieves superior performance compared to baseline methods that also leverage user-follow relationships, such as Alhosseini et al. and SATAR, with MCC improvements of 39.25% and 6.05%, respectively. This indicates that ESA-BotRGCN better exploits follow relationships by situating users within their social contexts.

## 5. Conclusions

To address the issue that existing social bot detection methods commonly overlook the semantic and emotional value of emojis, we innovatively propose ESA-BotRGCN, an emoji-driven end-to-end detection framework. This framework introduces an emoji–text enhancement strategy, combining GPT-4-based text coherence optimization and RoBERTa embeddings, effectively resolving the loss of emoji semantics and emotional value in traditional methods. Second, we design seven sentiment quantification metrics to systematically capture statistical differences in emotional expression patterns between bot and human accounts. Furthermore, an attention gating fusion mechanism is constructed to dynamically integrate diverse user features. Finally, by modeling heterogeneous social topologies with an RGCN, the detection robustness is substantially improved through the four aforementioned processes. Experiments on the TwiBot-20 benchmark dataset demonstrate that ESA-BotRGCN outperforms ten mainstream baseline models, highlighting the effectiveness of our emoji-driven multi-modal feature fusion and heterogeneous relationship modeling. Future work will focus on expanding multi-modal features by incorporating user-generated images, audio, and video content to enhance the model’s ability to identify camouflaged behaviors.

## Figures and Tables

**Table 1:** Examples of popular emoji and their text mapping.

Emoji	Text Mapping	Emoji	Text Mapping
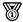	1st_Place_Medal	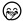	Face_Hand over Mouth
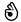	OK_Hand	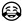	Face_Tears of Joy
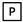	P_Button	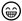	Face_Teeth Bared
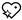	Sparkling_Heart	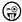	Winking Face_Tongue
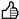	Thumbs_Up	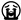	Crying Face

**Table 2:** User’s numerical features.

Feature Type	Feature Description
Emoji_avg_per_tweet	Number of emojis in each tweet
Emoji_variety_per_tweet	Variety of emojis in each tweet
Top_emoji_percentage	Proportion of the most commonly used emoji
URL_Usage_percentage	Proportion of tweets containing URLs
Followers_count	Number of account fans
Friends_count	Number of accounts concerned
Favourites_count	Number of likes
Statuses_counts	Number of tweets published by users
Active_days	Active days
Screen_name_length	Length of account name

**Table 3:** User’s categorical features.

Feature Type	Feature Description
protected	Whether the account is set as private
geo_enabled	Enable geographic location or not
verified	Whether the account is verified
contributors_enabled	Allow account sharing or not
is_translator	Translator or not
is_translation_enabled	Translation or not
profile_background_tile	Whether the background is tiled
profile_use_background_image	Whether the user uploads the background image
has_extended_profile	Whether there are extension files
default_profile	Whether to use the default theme background
default_profile_image	Is the default profile image being used

**Table 4:** Emoji preprocessing ablation study results.

Selected Tweets	Accuracy	F1-Score	MCC
Original tweets	0.8648	0.8793	0.7282
Emoji-to-text mapped tweet	0.8694	0.8839	0.7388
Emoji-to-text mapped and Coherence-improved tweet by Qwen-2.5	0.8731	0.8866	0.7429
Emoji-to-text mapped and Coherence-improved tweet by GPT-4	0.8746	0.8883	0.7468

**Table 5:** Sentiment feature ablation study results.

Selected Features	Accuracy	F1-Score	MCC
No sentiment feature	0.8639	0.8783	0.7263
Basic sentiment features	0.8664	0.8816	0.7322
Advanced sentiment features	0.8746	0.8883	0.7468

**Table 6:** GNN architecture selection ablation study results.

GNN Architecture	Accuracy	F1-Score	MCC
GAT	0.7413	0.7544	0.4824
GCN	0.7473	0.7666	0.4910
Neural Network	0.8605	0.8762	0.7200
ESA-BotRGCN	0.8746	0.8883	0.7468

**Table 7:** Attention mechanism ablation experiment results.

Experiment Configuration	Accuracy	F1-Score	MCC
No-attention mechanism	0.8564	0.8721	0.7147
Full-attention mechanism	0.8746	0.8883	0.7468

**Table 8:** Significance analysis of different feature settings.

Comparison Group	t-Value	*p*-Value	Significance
Emoji Only vs. Full ESA-BotRGCN	−8.09	0.000045	Significant
Sentiment Only vs. Full ESA-BotRGCN	−9.25	0.000099	Significant
No Sentiment and Emoji vs. Full ESA-BotRGCN	−16.8	0.00000	Extremely significant

**Table 9:** Bot detection performance on the TwiBot-20 benchmark.

Method	Accuracy	F1-Score	MCC
Lee et al. [51]	0.7456	0.7823	0.4879
Yang et al. [52]	0.8191	0.8546	0.6643
Kudugunta et al. [53]	0.8174	0.7517	0.6710
Wei et al. [54]	0.7126	0.7533	0.4193
Miller et al. [55]	0.4801	0.6266	−0.1372
Cresci et al. [27]	0.4793	0.1072	0.0839
Botometer [7]	0.5584	0.4892	0.1558
Alhosseini et al. [56]	0.6813	0.7318	0.3543
SATAR [57]	0.8412	0.8642	0.6863
BotRGCN [36]	0.8462	0.8707	0.7021
**ESA-BotRGCN**	**0.8746**	**0.8883**	**0.7468**

**Table 10:** Bot detection performance on the Cresci-15 benchmark.

Method	Accuracy	F1-Score	MCC
Botometer [7]	0.7259	0.7122	0.3159
Mazza et al. [58]	0.7200	0.7074	0.6868
RF-GNN [59]	0.9574	0.9547	0.9122
BotRGCN [36]	0.9452	0.9430	0.8927
DeeProBot [60]	0.8427	0.8559	0.8263
SCL [61]	0.8645	0.8868	0.8477
BotDCGC [40]	0.9334	0.9275	0.8861
**ESA-BotRGCN**	**0.9725**	**0.9783**	**0.9326**

## Data Availability

The original contributions presented in this study are included in the article. Further inquiries can be directed to the corresponding author.

## Abbreviations

The following abbreviations are used in this manuscript:

ESA-BotRGCN	Emoji-Driven Sentiment Analysis for Social Bot Detection with Relational Graph Convolutional Network
RGCN	Relational Graph Convolutional Network
MCC	Matthews Correlation Coefficient
CNNs	Convolutional Neural Networks
RNNs	Recurrent Neural Networks
GNN	graph neural network
URL	Uniform Resource Locator
LR	logistic regression
SVM	support vector machine
GB	gradient boosting
LCS	Longest Common Subsequence
LSTM	Long Short-Term Memory
BiGRU	bidirectional gated recurrent unit
UNK	unknown (placeholder for rare emojis)
MLPs	Multi-Layer Perceptrons
API	Application Programming Interface
TP	true positive
TN	true negative
FP	false positive
FN	false negative
BiLSTM	Bidirectional Long Short-Term Memory
GCNN	Graph Convolutional Neural Network
GAT	Graph Attention Network
GCN	Graph Convolutional Network
Adam	Adaptive Moment Estimation
Leaky-ReLU	Leaky Rectified Linear Unit
